# Supplementary material for: Synaptic vesicle endocytosis deficits underlie GBA-linked cognitive dysfunction in Parkinson’s disease and Dementia with Lewy bodies
Source: Res Sq. 2024 Dec 27:rs.3.rs-5649173. Preprint. [Version 1] doi: 10.21203/rs.3.rs-5649173/v1 (PMC11703330; doi:10.21203/rs.3.rs-5649173/v1)
Supplement: 1 [file NIHPPRS5649173V1-supplement-1.pdf]

Supplementary Files

This is a list of supplementary files associated with this preprint. Click to download.

- SupplTable1DEGlistGbavsWTVidyadharaetal.2024.xlsx
- SupplTable2DEGlistGbaSNCAvsWTVidyadharaetal.2024.xlsx
- SupplTable3DEGlistSNCAvsWTVidyadharaetal.2024.xlsx
- SupplTable4DEGsdrivingGOpathwaysVidyadharaetal.2024.xlsx
- SupplementalinformationVidyadharaetal.2024.pdf
